# Microsurgical Resection of Unruptured Brain Arteriovenous Malformations: A 12-Year Single-Center Experience

**DOI:** 10.3390/medicina61111993

**Published:** 2025-11-06

**Authors:** Aleksandar Janicijevic, Nikola Repac, Stefan Mandic-Rajcevic, Jelena Kostić, Nikola Jovicevic, Aleksandar Milosavljevic, Milica Cancarevic Janicijevic, Dean Vidovic, Vladimir Jovanovic, Goran Tasic

**Affiliations:** 1 Clinic of Neurosurgery, University Clinical Center of Serbia, 11000 Belgrade, Serbia; aleksandarjanicijevic82@gmail.com (A.J.); nyjaphen@yahoo.com (N.R.); n.jovicevic05@gmail.com (N.J.);; 2 Faculty of Medicine, University of Belgrade, 11000 Belgrade, Serbia; stefan.mandic-rajcevic@med.bg.ac.rs; 3 Clinic of Neurosurgery, University Clinical Center of Kragujevac, 34000 Kragujevac, Serbia; 4 Faculty of Medical Science, University of Kragujevac, 34000 Kragujevac, Serbia; 5 Special Hospital for Cerebrovascular Diseases “Sveti Sava”, 11000 Belgrade, Serbia; 6 General Hospital Doboj, 74000 Doboj, Republika Srpsa, Bosnia and Herzegovina; dean.vidovic1990@gmail.com

**Keywords:** brain arteriovenous malformation, unruptured AVM, microsurgery, supplementary Spetzler–Martin grading, epilepsy, surgical outcomes

## Abstract

*Background and Objectives*: The management of unruptured brain arteriovenous malformations (ubAVMs) remains controversial, particularly following the ARUBA trial, which favored conservative management. However, concerns regarding the validity of its findings persist. This study aimed to evaluate the outcomes of microsurgical resection of ubAVMs in a high-volume neurosurgical center. *Materials and Methods*: This is a retrospective single-center study that analyzed 52 patients treated by microsurgical resection at the Cerebrovascular Department, University Clinical Center of Serbia, between January 2010 and January 2022. All patients were classified according to the supplementary Spetzler–Martin (suppl-SM) grading system and stratified into low-risk (suppl-SM ≤ 6) and high-risk (suppl-SM > 6) groups. Functional outcomes were assessed using the modified Rankin Scale (mRS) at discharge and 9-month follow-up. *Results*: The mean patient age was 38.8 years, with equal gender distribution. Epileptic seizure was the most common presenting symptom (80.4%). In the low-risk group, there were no deaths, and poor outcomes were rare (6.8% at discharge; 2.3% at 9 months). Conversely, the high-risk group demonstrated significantly worse outcomes (62.5% poor outcome at discharge, 28.6% at 9 months). The overall hemorrhagic stroke rate was 5.8%, with one fatality (12.5%) in the high-risk subgroup. Absence of superficial venous drainage and presence of combined/deep venous drainage were strongly associated with poor outcomes. *Conclusions*: Microsurgical resection of ubAVMs is a safe and effective treatment strategy for low-grade lesions, yielding excellent functional outcomes and minimal morbidity. Our findings, supported by other large series, reinforce microsurgery as the gold standard for low-grade ubAVMs in appropriately selected patients.

## 1. Introduction

Brain arteriovenous malformations (bAVMs) are complex vascular lesions that are most commonly diagnosed in younger and working-age populations. Although considered relatively rare, with an estimated prevalence of 10–18 per 100,000 adults and an incidence of 1 case per 100,000 patients per year, AVMs account for approximately 2% of all hemorrhagic strokes [1]. Their clinical presentation usually manifests as either intracranial hemorrhage or epileptic seizures. The annual risk of hemorrhage from unruptured AVMs (ubAVMs) is estimated to be between 2.1% and 4.12%, while the cumulative risk over a 20-year period reaches approximately 29% [2,3]. Hemorrhage from these lesions can lead to significant morbidity and mortality, with reported mortality rates ranging from 12% to 66.7%, and permanent neurological deficits persisting in 23% to 40% of patients [4,5]. Considering the high rates of morbidity and mortality following hemorrhage, accurate risk assessment and proper treatment selection in patients with unruptured bAVMs are of critical importance.

While there is general agreement that ruptured bAVMs require aggressive treatment to prevent rebleeding, the optimal management of unruptured brain arteriovenous malformations (ubAVMs) remains controversial, especially following the results of the ARUBA trial [6]. This study found that patients managed conservatively had lower rates of mortality and stroke compared to those who received interventional therapy. Specifically, over a mean follow-up of 33.2 months, 30.7% of patients in the intervention group experienced symptomatic stroke or death compared to only 10.1% in the medical management group. Furthermore, clinical impairment (modified Rankin Scale score ≥ 2) was more common in the intervention group (46.2%) than in the conservative group (15.1%). The annual rate of spontaneous bAVM rupture was 2.2%.

However, the ARUBA trial has several important limitations. Only 226 out of 1740 eligible patients (13%) were randomized, raising concerns about selection bias. Of those assigned to intervention, most had low-grade bAVMs, but only a small proportion (15.8%) underwent surgical resection, which is considered the gold standard for low-grade lesions. Instead, many patients received embolization alone or stereotactic radiosurgery, treatments that may carry a higher risk of hemorrhage.

Since ARUBA, many authors have criticized the study’s design, short follow-up, and conclusions. Subsequent studies focusing on surgical management of ARUBA-eligible patients have reported lower rates of symptomatic stroke or death (9.3–16.1%) and clinical impairment (4.5–13.8%) than those seen in the ARUBA intervention group [7,8,9,10].

In this paper, we present our own experience with the surgical treatment of unruptured brain arteriovenous malformations.

## 2. Materials and Methods

This single-center retrospective study included 52 patients with diagnosed unruptured brain AVMs (ubAVMs) treated at the Cerebrovascular Department, Clinic for Neurosurgery, University Clinical Center of Serbia, between January 2010 and January 2022.

All cases of ubAVM were evaluated by a multidisciplinary neurovascular board comprising a vascular neurosurgeon, interventional neuroradiologist, and radiation oncologist. Treatment options—including conservative management, microsurgical excision, stereotactic radiosurgery (SRS), endovascular embolization, and multimodal approaches—were considered individually.

In general, ubAVMs with a supplemented Spetzler–Martin (Suppl. SM) grade ≤ 6 were primarily treated with microsurgery. Higher-grade lesions were managed on a case-by-case basis depending on factors such as size, location, nidus compactness, and eloquence of surrounding brain tissue. Small, deep AVMs (e.g., thalamus, basal ganglia, and brainstem) were typically selected for SRS. Suppl. SM grade 9 and 10 lesions were mostly managed conservatively, especially in older patients or those with significant comorbidities.

Patients eligible for inclusion were those with unruptured AVMs who underwent microsurgical resection alone. Exclusion criteria included patients with other congenital vascular lesions such as vein of Galen malformations, dural arteriovenous fistulas, cavernous malformations, and venous malformations. The AVM diagnosis was confirmed by postoperative histopathological examination. Collected data included demographic characteristics of the patients (sex and age), presenting symptoms, and morphological features of the arteriovenous malformations observed on CT angiography and DS angiography (location, nidus size, vascular supply territory, type of venous drainage, and presence of venous ectasia). All bAVMs were classified using the supplemented Spetzler–Martin (Supp-SM) grading system and divided into two groups: Supp-SM ≤ 6 (low-risk group) and Supp -SM > 6 (high-risk group). Functional status was assessed using the modified Rankin Scale (mRS) in the immediate postoperative period (until hospital discharge) and at the 9-month follow-up. Based on mRS scores, patients were categorized into two outcome groups: mRS ≤ 2 (good outcome) and mRS > 2 (poor outcome).

### Statistical Analysis

All patient data were stratified by risk category (low vs. high). Descriptive statistics were generated for all variables; continuous data such as age were presented as mean (standard deviation), while categorical data were summarized as absolute frequencies (n) and percentages (%). To assess differences between the low- and high-risk groups, inferential statistical tests were applied. The choice of test was determined by the nature of the variable:

- Continuous Data: Differences in normally distributed continuous variables were assessed using Student’s *t*-test or the Mann–Whitney U test.
- Nominal/Dichotomous Data: Comparisons of nominal categorical variables (e.g., AVM location laterality and arterial supply presence/absence) were performed using Pearson’s Chi-square test. Fisher’s exact test was used when the expected cell counts were less than five. A two-sided *p*-value less than 0.05 was considered the threshold for statistical significance in all analyses. Statistical analysis was performed using R statistical software (R version 4.3.1) (2023-06-16 ucrt) “Beagle Scouts” with the compare Groups package.

## 3. Results

Table 1 presents the patient and AVM anatomical characteristics by risk group. The patient cohort consisted of 52 individuals with a mean age of 38.8 years and a slight male predominance (51.9%). The most frequently observed arteriovenous malformation (AVM) size was in the 3–6 cm range, accounting for 65.4% of all cases, and the majority of patients (71.2%) presented with superficial venous drainage. Stratification by risk group revealed significant anatomical distinctions. Although age was comparable between groups, a notable gender disparity was observed: males constituted 87.5% of the high-risk group versus 45.5% of the low-risk group (*p* = 0.051). AVM size was a key delineating factor, as 100.0% of high-risk patients had AVMs between 3 and 6 cm. The most statistically significant differences were found in the venous architecture. The absence of superficial venous drainage was a predominant feature in the high-risk group (87.5%) but was infrequent in the low-risk group (18.2%) (*p* < 0.001). Furthermore, the presence of combined venous drainage was significantly more common in the high-risk cohort (75.0%) compared to the low-risk cohort (9.1%) (*p* < 0.001).

Table 2 presents the clinical characteristics of patients by risk. In terms of clinical presentation for the entire cohort, epilepsy was the most prevalent symptom, observed in 80.4% of patients with available data. Other presenting symptoms such as headache (23.1%) and neurological deficits (19.2%) were less common. When comparing the low-risk and high-risk groups, the overall prevalence of these conditions did not demonstrate statistically significant differences. The incidence of any epilepsy was similar between low-risk (79.1%) and high-risk (87.5%) patients (*p* = 1.000). However, a non-significant trend was noted regarding seizure type. Partial epilepsy was more than twice as frequent in the high-risk group (50.0%) as in the low-risk group (20.5%) (*p* = 0.096). Conversely, generalized tonic–clonic seizures were more commonly reported among low-risk patients (62.8%) than high-risk patients (37.5%), although this difference was not statistically significant (*p* = 0.249).

An evaluation of perioperative characteristics (Table 3) for all 52 participants indicated that postoperative complications were infrequent; intracerebral hemorrhage occurred in 5.8% of cases and meningitis in 7.7%. Functional outcomes, assessed via the modified Rankin (mRS) score, showed that 15.4% of all patients had a poor outcome at discharge, which improved to 5.9% at the 9-month follow-up. However, these aggregate figures obscure a statistically significant disparity between the risk groups. While complication rates were comparable, functional outcomes were profoundly different. In the low-risk group, a poor outcome was rare, recorded in only 6.8% of patients at discharge and 2.3% at 9 months. In contrast, the high-risk group exhibited significantly poorer outcomes, with 62.5% having a poor outcome at discharge (*p* = 0.001) and 28.6% remaining with a poor outcome after 9 months (*p* = 0.046). The rate of functional improvement between discharge and the 9-month follow-up did not differ significantly between the groups.

**Figure 1:**
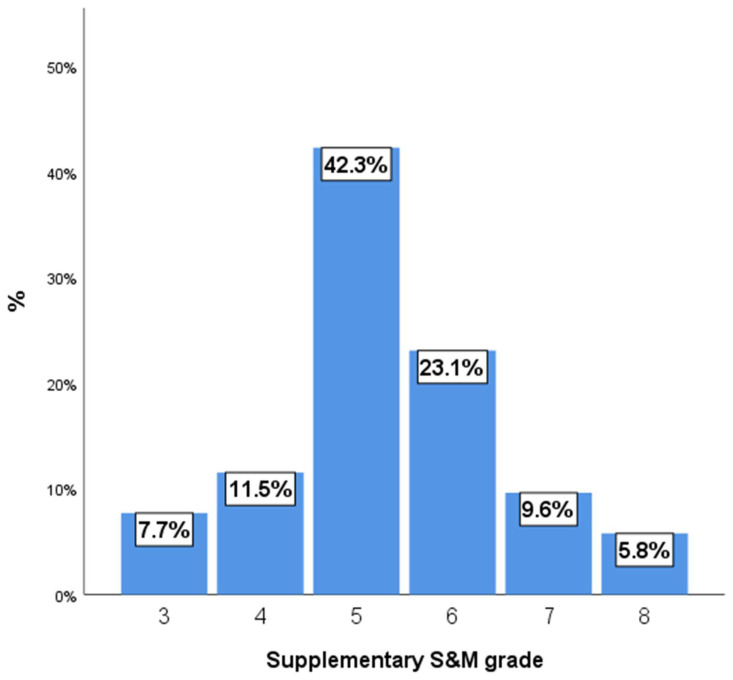
Supplementary S&M grade.

Figure 1 shows the supplementary Spetzler–Martin (S&M) grade distribution, which was skewed toward higher-grade AVMs. The most common grade was 5, accounting for 42.3% of the AVMs, followed by grade 6 at 23.1%. The distribution then decreased, with grades 7 and 8 making up 9.6% and 5.8%, respectively. Grades 3 and 4 represented the minority, at 7.7% and 11.5%.

The analysis of the localization of AVMs in our case series revealed a consistent and significant lateralization to the left cerebral hemisphere. A strong left-sided predominance was observed in AVMs located in the frontal (N = 15, 86.7% left) and parietal lobes (N = 11, 81.8% left). This pattern of left-sided predilection was also evident in motor cortex AVMs (N = 8, 75.0% left), as well as in temporal (N = 10, 70.0% left) and occipital AVMs (N = 7, 57.1% left). Although less frequent in our cohort, even the deep (N = 3, 66.7% left), cerebellar (N = 1, 100% left), and ventricular AVMs (N = 3, 100% left) followed a similar trend. A total of 22 AVMs were supplied by the Anterior Cerebral Artery (ACA), demonstrating a perfectly balanced distribution with 50.0% of cases fed by the left ACA and 50.0% by the right. The Middle Cerebral Artery (MCA), which was the most common feeder (N = 30), showed a slight left-sided preference, supplying 56.7% of AVMs on the left and 43.3% on the right. A clear left-sided predominance was also observed in the Pericallosal Artery, which supplied 63.6% of its AVMs on the left. The Posterior Communicating Artery (PCoA) showed a right-sided predominance, supplying 60.0% of its AVMs from the right side. The limited data for the Anterior Choroidal Artery (AChA), Anterior Communicating Artery (ACoP), and the Posterior and Anterior Inferior Cerebellar Arteries (PICA and AICA) indicated an exclusive right-sided supply. These findings suggest that while major feeding vessels like the ACA maintain a symmetrical supply, other specific arteries may exhibit a distinct lateralization in their contribution to AVM blood flow.

**Figure 2:**
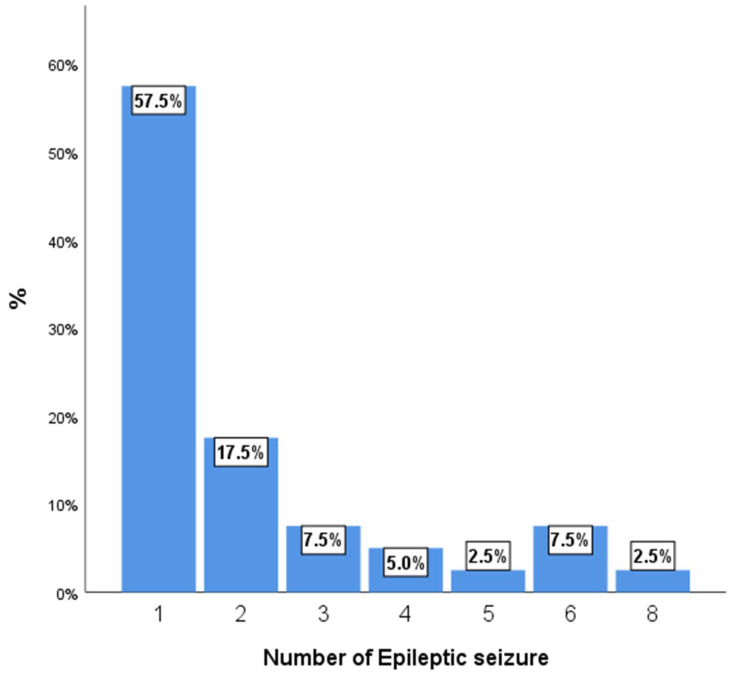
Number of epileptic seizures per patient.

Figure 2 shows the number of epileptic seizures per patient. The most frequent clinical presentation was epilepsy, with the majority of patients experiencing a low number of epileptic seizures. A significant portion of the cohort, 57.5%, had only one seizure, while an additional 17.5% experienced two seizures. A smaller proportion of patients had three or more seizures, with the distribution tapering off for higher numbers of events.

Table 4 shows a comparison of perioperative functional outcomes, as measured by the modified Rankin Scale (mRS) score at discharge and 9 months after discharge, between two patient groups: a low-risk cohort (N = 44) and a high-risk cohort (N = 8). The analysis revealed a statistically significant difference in outcomes between the two groups at discharge (*p* < 0.001). The majority of the low-risk cohort achieved favorable functional outcomes, with 22 patients (50.0%) discharged with an mRS score of 1 and an additional 19 patients (43.2%) with a score of 2. In contrast, no patients in the high-risk cohort achieved an mRS score of 1, and only three patients (37.5%) were discharged with a score of 2. The high-risk group demonstrated a greater prevalence of severe morbidity, with three patients (37.5%) having an mRS score of 4 and one patient experiencing a fatal outcome (mRS score of 6).

Nine months after discharge, a substantial majority of patients in the low-risk group achieved favorable functional outcomes, with 15 patients (34.1%) reporting a perfect mRS score of 0 and 25 patients (56.8%) achieving a score of 1. No patients in the high-risk cohort achieved a perfect score of 0. While two patients (28.6%) from the high-risk group reached an mRS score of 1, the majority of the cohort had less favorable outcomes, with three patients (42.9%) scoring 2 and two patients (28.6%) scoring 3.

**Figure 3:**
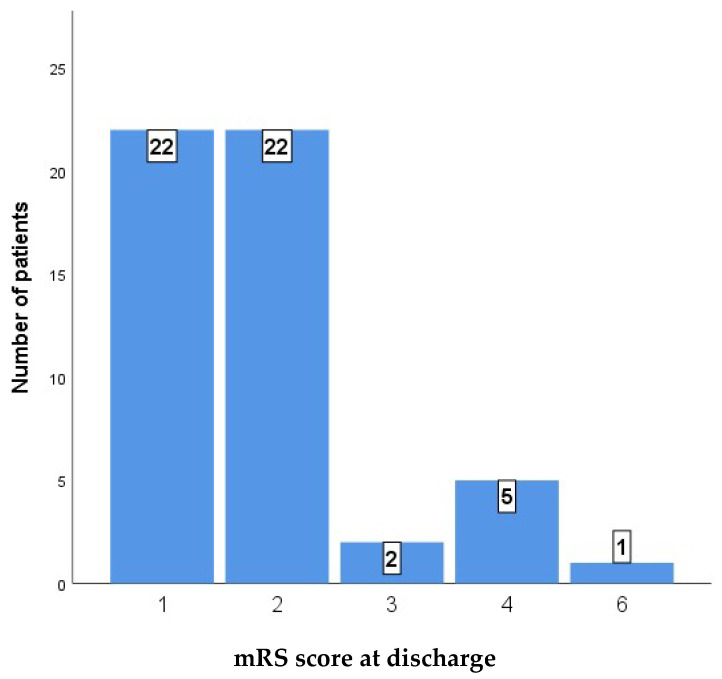
mRS score at discharge.

**Figure 4:**
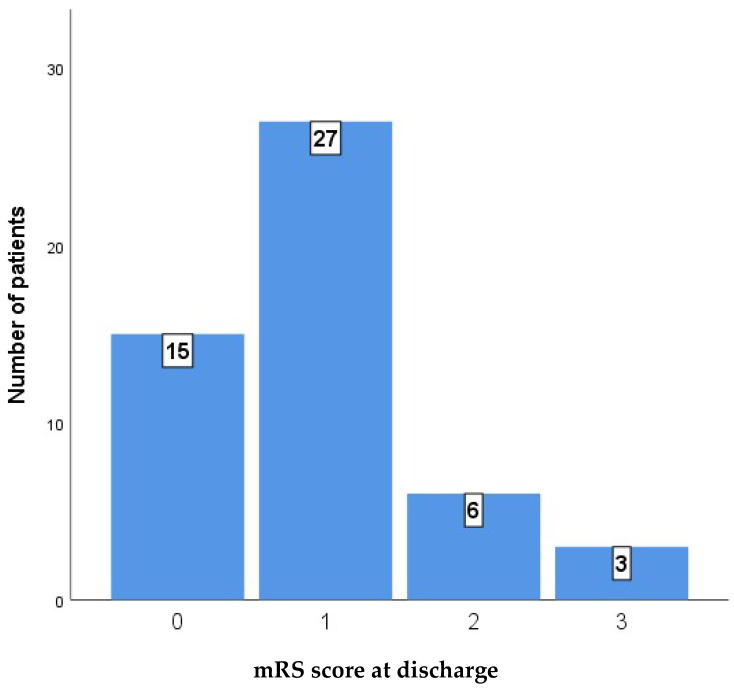
mRS score after 9 months.

Figure 3 and Figure 4 show the distribution of outcomes as measured by the mRS in the whole case series at discharge and at 9 months after discharge.

## 4. Discussion

This retrospective cohort study aimed to evaluate functional outcomes in patients with unruptured brain arteriovenous malformations treated by microsurgical resection as well as clinical and radiological characteristics of AVM. We found that low-grade AVMs were linked to favorable functional outcomes, epilepsy was the most frequent presentation, and venous drainage patterns were strongly associated with surgical risk. Our patient demographics were consistent with those reported in the recent literature, supporting the generalizability of our findings.

All unruptured brain AVMs in our cohort were treated by surgical resection, which we consider the gold standard of treatment, particularly for low-grade AVMs. Our position is supported by findings from other authors [11,12,13]. In our study, all surgeries were performed without prior embolization, which is in line with the conclusions of a meta-analysis published in 2023 [14]. This analysis included 32 studies and compared outcomes in 1088 patients who underwent surgery with preoperative embolization and 1828 patients operated on without prior embolization. It demonstrated no statistically significant differences in AVM obliteration, mortality, complications, worse mRS, or intraoperative blood loss between the two groups.

In our study, all unruptured brain AVMs were classified as either low-risk (Supp. SM ≤ 6) or high-risk (supp. SM > 6) using the supplementary Spetzler–Martin (Supp. SM) scale [15]. The cut-off score of 6 was chosen based on the multicenter study by Kim et al., who validated the supplemented Spetzler–Martin grading system on 1009 patients and showed that patients with scores ≤ 6 had acceptable surgical risk and favorable outcomes, while higher scores were linked to increased morbidity and mortality [16].

In our study, microsurgical resection of unruptured brain arteriovenous malformations (ubAVMs) demonstrated high safety and efficacy, particularly among low-grade lesions. Of all 52 patients, 1 patient died and 3 experienced postoperative hemorrhagic stroke, corresponding to an overall mortality rate of 1.9% and a hemorrhagic stroke rate of 5.8%. These complications were more frequent in the high-grade group (12.5%) compared to the low-grade group (4.5%), with one fatal outcome (12.5%) occurring in the high-risk subgroup. Notably, no deaths were recorded among low-grade AVMs. Numerous studies have demonstrated that surgical treatment of low-grade brain arteriovenous malformations (AVMs) is associated with favorable outcomes and low mortality rates. Link and colleagues published a single-center study involving 86 patients with unruptured AVMs. In the subgroup of patients treated microsurgically, there were no deaths, and the rate of hemorrhagic stroke was 6% [10]. Additionally, Javadpour et al. reported a series of 34 surgically treated patients with unruptured AVMs in which no deaths or postoperative hemorrhagic strokes occurred [7]. This further underscores the safety of microsurgical resection for unruptured AVMs when performed in high-level, specialized centers. Likewise, Schramm et al. observed that in a cohort of 288 AVM patients, of whom 144 had unruptured lesions treated surgically, the mortality rate was 0.7%. In the subgroup of 78 low-grade AVMs, no deaths were reported, which is consistent with the findings of our study [8]. In another prospective series of 282 surgically treated unruptured AVMs (published in 2017), the overall rate of mortality was 1.1%, and that of hemorrhagic stroke was 1.8%. In the subgroup of low-grade AVMs, there were no deaths and no hemorrhagic stroke cases [17]. In their series, which included 155 surgically treated patients, Wong et al. reported no mortality and a hemorrhagic stroke rate of 3.9%. In the low-grade subgroup of 118 patients, hemorrhagic stroke occurred in two patients (1.7%) [9].

In our series, the overall poor outcome was 15.3% at discharge and 5.9% after nine months of follow-up. Link et al. reported overall early minor and major postoperative deficits in 12.8% and 5.8% of cases, respectively, with only 4.5% showing permanent neurological impairment after six months [10]. Similarly, Javadpour et al. observed 17.6% new neurological deficits overall, with the majority being transient, and 94% of patients achieved excellent outcomes (mRS 0–1) at the six-month follow-up [7]. Likewise, in Schramm et al.’s study, overall early deficit occurred in 16.7%, and significant permanent neurological deficits occurred in 7.6% of all cases [8]. Tong et al. reported overall poor outcome at last follow-up in 3.5% of cases in their series [17]. Wong et al. reported overall early disabling and permanent disabling deficits in 12.3% and 4.5% of cases, respectively, in their series [9].

Functional outcomes differed significantly between the low-risk and high-risk groups in our study. Among low-grade cases (Supp. SM ≤ 6), poor outcomes (mRS ≥ 3) were rare—6.8% at discharge and only 2.3% at nine months. In contrast, high-grade AVMs (Supp. SM > 6) were associated with significantly worse results, with 62.5% of patients showing poor outcomes at discharge and 28.6% at nine months (*p* = 0.001), confirming a strong relationship between surgical risk and lesion complexity. A slightly higher proportion of poor outcomes observed in the high-grade subgroup (Supp. SM > 6) in our study can be explained by the composition of our high-risk cohort, in which three out of eight patients (37.5%) had lesions graded as supplemented Spetzler–Martin (Supp. SM) 8. Such cases represent the upper end of the high-grade spectrum and are known to carry substantially higher operative risk. These results support the validity and clinical utility of the supplemented Spetzler–Martin grading system, consistent with previous external validation studies [16,18]. Link et al. reported, in the low-grade AVM subgroup, early minor and major postoperative deficits in 7.8% and 3.9% of cases, respectively. Only 2% of cases showed permanent neurological impairment after six months, which is consistent with the findings of our study. In the high-grade AVM group, rates of early minor and major postoperative deficit were 5.7% and 8.6%, respectively, and permanent neurological deficit after six months was reported in 5.7% cases [10]. Tong et al., in their series, which involved a subgroup of 186 patients with low-grade AVM, reported poor outcomes (mRS > 2) in 4 patients (2.2%), and in a higher-grade AVM subgroup of 96 patients, poor outcomes were reported in 13 patients (13.5%) [17]. Schramm et al. reported, in their subgroup of 78 low-grade unruptured AVMs, early postoperative deficit in 12.8% and permanent deficit in 2.6% of cases [8]. In Wong et al.’s study, 118 of 155 unruptured brain AVMs were low-grade AVMs. In the low-grade subgroup, they reported early neurological deficit in 9.3% patients and permanent neurological deficit in 3.4% at the last follow-up [9].

Our results, together with those from other large studies, differ with the findings of the ARUBA trial, which concluded that conservative medical management was superior to interventional treatment. In the ARUBA intervention group, 30.7% of patients experienced symptomatic stroke or death, compared to only 10.1% in the medical management group, over a median follow-up period of 33.2 months. The intervention group also had a mortality rate of 2.6% and a hemorrhagic stroke rate of 21.9%. The poor outcomes observed in the ARUBA trial have been attributed to significant methodological and treatment-related limitations. Only 226 out of 1740 eligible patients (13%) were randomized, raising concerns about selection bias. Of those assigned to intervention, most had low-grade AVMs (68%), but only a small proportion (15.8%) underwent surgical resection, which is considered the gold standard for low-grade lesions. Instead, most received only partial treatment with embolization and/or radiosurgery, including 30 patients (26.3%) who were treated solely with embolization—a method previously linked to a higher risk of hemorrhage. Furthermore, the average follow-up period of less than three years is insufficient to draw reliable conclusions regarding the long-term risk of hemorrhage in conservatively managed patients [19,20,21,22].

In our cohort, epileptic seizures were the most frequent initial clinical manifestation, whereas headaches and focal neurological deficits were less commonly observed. This is consistent with the findings of Zhao et al. [23], who reported seizures as the initial presentation in 56.8% of 176 patients with unruptured AVMs. Similarly, Garcin et al. [24] also identified epilepsy as a predominant presenting symptom in a significant proportion of AVM cases. Their study also suggested that seizures are more likely to occur in AVMs with superficial venous drainage. The unusually high proportion of patients in our cohort presenting with epileptic seizures (80.4%), may be attributed to several contributing factors. First, a high proportion of AVMs in our cohort (71.2%) exhibited superficial venous drainage, a factor previously shown to be significantly associated with seizure onset. Second, our cohort consisted predominantly of younger adults (mean age 38.8 years), a population in which seizures are a more common initial manifestation of AVMs than in older patients [25,26,27]. Third, selection bias likely played a role, as patients experiencing seizures are more frequently referred for neurosurgical evaluation and intervention and are thus more likely to be included in a surgical cohort. Finally, limitations inherent to retrospective data collection may have resulted in underreporting of less specific symptoms, such as headache or cognitive changes, while seizures, being more overt and clinically significant, were consistently documented. Interestingly, some historical analyses have proposed that Julius Caesar may have suffered from epilepsy secondary to an underlying brain arteriovenous malformation based on descriptions of his episodic symptoms in later life [28].

Venous drainage patterns emerged as a key prognostic factor. In our study, the absence of superficial venous drainage emerged as a dominant feature in the high-risk group (87.5%), whereas it was considerably less frequent in the low-risk group (18.2%). Additionally, the presence of combined venous drainage was significantly more prevalent in the high-risk cohort (75.0%) compared to the low-risk cohort (9.1%). These findings are consistent with previous multivariate analyses that identify deep venous drainage as one of the key prognostic factors for unfavorable outcomes [23,29]. Additionally, the type of venous drainage has been recognized within existing prognostic scoring systems as an important parameter for predicting poor outcomes, including both the original Spetzler–Martin grading system and the supplementary Spetzler–Martin scale [15,30].

The demographic characteristics of our patients (mean age 38.8 years and equal gender distribution) are comparable to recent series in the literature, which report the most common presentation in the young adult population [29].

The predominant supratentorial localization and the rare occurrence of vertebrobasilar AVMs in our sample are consistent with data from larger cohorts [3,23].

## 5. Conclusions

Our study confirms that microsurgical resection of unruptured brain arteriovenous malformations (AVMs) without prior embolization is a safe and effective treatment option for low-grade AVMs with exceptionally low rates of mortality and permanent neurological deficits. The presence of combined or deep venous drainage emerged as an important prognostic factor for poor outcomes, in accordance with established validated prognostic grading systems. The main limitation of this study lies in its relatively short follow-up duration of one year. Therefore, future multicentric investigations encompassing a broader network of hospitals across Eastern Europe are recommended to provide more comprehensive data and to further improve patient survival outcomes.

Despite limitations, the strength of this work lies in the relatively large cohort of surgically treated patients, the homogeneous therapeutic approach (exclusive microsurgical resection), and the systematic evaluation of functional outcomes within a controlled timeframe. Our results, together with those of numerous other studies, further reinforce the role of microsurgery as the gold standard in the treatment of low-grade unruptured AVMs, especially in high-volume centers. Future studies should focus on refining risk stratification and optimizing management strategies for high-grade lesions.

## Figures and Tables

**Table 1:** Patients and anatomical characteristics of AVM by risk.

	All Patients	Low Risk	High Risk	*p*-Value
	*N* = 52	*N* = 44	*N* = 8	
Age (years)	38.8 (17.1)	37.6 (16.7)	45.1 (19.0)	0.322
Gender:				0.051
Male	27 (51.9%)	20 (45.5%)	7 (87.5%)	
Female	25 (48.1%)	24 (54.5%)	1 (12.5%)	
AVM size category:				/
<3	16 (30.8%)	16 (36.4%)	0 (0.0%)	
3–6	34 (65.4%)	26 (59.1%)	8 (100.0%)	
>6	2 (3.8%)	2 (4.5%)	0 (0.0%)	
Side of AVM:				0.134
Left	27 (51.9%)	25 (56.8%)	2 (25.0%)	
Right	25 (48.1%)	19 (43.2%)	6 (75.0%)	
Superficial venous drainage:				<0.001
No	15 (28.8%)	8 (18.2%)	7 (87.5%)	
Yes	37 (71.2%)	36 (81.8%)	1 (12.5%)	
Deep venous drainage:				1.000
No	47 (90.4%)	40 (90.9%)	7 (87.5%)	
Yes	5 (9.6%)	4 (9.1%)	1 (12.5%)	
Combined venous drainage:				<0.001
No	42 (80.8%)	40 (90.9%)	2 (25.0%)	
Yes	10 (19.2%)	4 (9.1%)	6 (75.0%)	
Presence of venous ectasia:				0.122
No	21 (40.4%)	20 (45.5%)	1 (12.5%)	
Yes	31 (59.6%)	24 (54.5%)	7 (87.5%)	

**Table 2:** Clinical presentation by risk.

	All Patients	Low Risk	High Risk	*p*-Value
	*N* = 52	*N* = 44	*N* = 8	
Epilepsy partial:				0.096
No	39 (75.0%)	35 (79.5%)	4 (50.0%)	
Yes	13 (25.0%)	9 (20.5%)	4 (50.0%)	
Epilepsy GTC:				0.249
No	21 (41.2%)	16 (37.2%)	5 (62.5%)	
Yes	30 (58.8%)	27 (62.8%)	3 (37.5%)	
Epilepsy:				1.000
No	10 (19.6%)	9 (20.9%)	1 (12.5%)	
Yes	41 (80.4%)	34 (79.1%)	7 (87.5%)	
Headache:				1.000
No	40 (76.9%)	34 (77.3%)	6 (75.0%)	
Yes	12 (23.1%)	10 (22.7%)	2 (25.0%)	
Neurological deficit:				0.642
No	42 (80.8%)	36 (81.8%)	6 (75.0%)	
Yes	10 (19.2%)	8 (18.2%)	2 (25.0%)	

**Table 3:** Perioperative characteristics and outcomes by risk.

	All Participants	Low Risk	High Risk	*p*-Value
	*N* = 52	*N* = 44	*N* = 8	
Complication intracerebral hemorrhage:				0.401
No	49 (94.2%)	42 (95.5%)	7 (87.5%)	
Yes	3 (5.8%)	2 (4.5%)	1 (12.5%)	
Complication meningitis:				0.499
No	48 (92.3%)	41 (93.2%)	7 (87.5%)	
Yes	4 (7.7%)	3 (6.8%)	1 (12.5%)	
Complication wound infection:				/
No	50 (96.2%)	42 (95.5%)	8 (100.0%)	
Yes	2 (3.8%)	2 (4.5%)	0 (0.0%)	
mRS score at discharge:				0.001
Bad outcome	8 (15.4%)	3 (6.8%)	5 (62.5%)	
Good outcome	44 (84.6%)	41 (93.2%)	3 (37.5%)	
mRS score after 9 months:				0.046
Bad outcome	3 (5.9%)	1 (2.3%)	2 (28.6%)	
Good outcome	48 (94.1%)	43 (97.7%)	5 (71.4%)	
mRS Improvement:				0.457
No	47 (92.2%)	41 (93.2%)	6 (85.7%)	
Yes	4 (7.8%)	3 (6.8%)	1 (14.3%)	

**Table 4:** Perioperative characteristics and outcomes by risk.

	All Participants	Low Risk	High Risk	*p* Value
	*N* = 52	*N* = 44	*N* = 8	
mRS score at discharge:				<0.001
1	22 (42.3%)	22 (50.0%)	0 (0.0%)	
2	22 (42.3%)	19 (43.2%)	3 (37.5%)	
3	2 (3.8%)	1 (2.3%)	1 (12.5%)	
4	5 (9.6%)	2 (4.5%)	3 (37.5%)	
6	1 (1.9%)	0 (0.0%)	1 (12.5%)	
mRS score after 9 months:				/
0	15 (29.4%)	15 (34.1%)	0 (0.0%)	
1	27 (52.9%)	25 (56.8%)	2 (28.6%)	
2	6 (11.8%)	3 (6.8%)	3 (42.9%)	
3	3 (5.9%)	1 (2.3%)	2 (28.6%)	

## Data Availability

The data supporting the findings of this study are available from the corresponding author upon reasonable request.

## Abbreviations

The following abbreviations are used in this manuscript:

AVM	Arteriovenous Malformation
bAVM	Brain Arteriovenous Malformation
ubAVM	Unruptured Brain Arteriovenous Malformation
mRS	Modified Rankin Scale
CT	Computed Tomography
DSA	Digital Subtraction Angiography
suppl. S-M	Supplementary Spetzler–Martin (grading system)
